# Effects of narasin supplementation on dry matter intake and rumen fermentation characteristics of *Bos indicus* steers fed a high-forage diet

**DOI:** 10.1093/tas/txz164

**Published:** 2019-10-16

**Authors:** Daniel M Polizel, Bruno I Cappellozza, Fernanda Hoe, Catarina N Lopes, José Paulo Barroso, Alexandre Miszura, Gabriela B Oliveira, Luiz Gobato, Alexandre V Pires

**Affiliations:** 1 Department of Nutrition and Animal Production, College of Veterinary and Animal Science, University of São Paulo, Pirassununga, SP, Brazil; 2 Elanco Animal Health, São Paulo, SP, Brazil; 3 Department of Animal Sciences, University of São Paulo, Piracicaba, SP, Brazil

**Keywords:** digestibility, dry matter intake, forage, narasin, volatile fatty acids

## Abstract

This study evaluated the effects of narasin on intake and rumen fermentation characteristics of *Bos indicus* steers offered a high-forage diet for 140 d. On day 0 of the study, 30 rumen-fistulated Nellore steers [initial body weight (BW) = 281 ± 21 kg] were assigned to 30 individual pens in a randomized complete block design according to their initial BW. Animals were randomly assigned to 1 of the 3 treatments: 1) forage-based diet without narasin (CONT; *n* = 10), 2) CONT diet plus 13 ppm of narasin (13NAR; *n* = 10), and 3) CONT diet plus 20 ppm of narasin (20NAR; *n* = 10). The forage used was Tifton-85 (*Cynodon dactylon* spp.), whereas the carrier for narasin was a 50:50 mixture of soybean hull:corn. The experimental period was divided into 5 periods of 28 d each. Throughout the experimental period, total dry matter intake (DMI) was recorded daily, whereas mineral salt intake was recorded weekly. Blood and ruminal fluid samples were collected on day 0 (prior to treatment feeding), 28, 56, 84, 112, and 140 of the study. Moreover, total tract apparent nutrient digestibility was performed for a 5-d period every 28 d. No treatment effects were observed on forage, mineral, concentrate, or total DMI (*P* ≥ 0.22). Nonetheless, 13NAR tended to have a greater mineral intake vs. 20NAR cohorts (*P* = 0.08) Narasin-supplemented animals had reduced rumen acetate, Ac:Pr ratio, as well as greater (*P* ≤ 0.02) rumen propionate concentrations vs. CONT cohorts. Moreover, 13NAR increased rumen propionate and decreased butyrate, Ac:Pr vs. 20NAR cohorts (*P* ≤ 0.01). Throughout the experimental period, narasin-supplemented animals had reduced ammonia concentrations vs. CONT cohorts (*P* < 0.01), whereas no differences were observed between 13NAR and 20NAR (*P* = 0.80). No treatment or dose effects were observed (*P* ≥ 0.23) on DM, organic matter (OM), protein, neutral detergent fiber (NDF), acid detergent fiber (ADF), and mineral digestibility. Animals fed 13NAR had a reduced mean plasma urea concentration vs. CONT cohorts (*P* = 0.03), whereas no further differences were observed (*P* ≥ 0.12). In summary, narasin supplementation to beef steers offered a high-forage diet did not impact forage, mineral, and total DMI, as well as nutrient digestibility, whereas rumen fermentation characteristics, rumen ammonia, and plasma urea concentrations were positively impacted and lasted throughout the experimental period. Additionally, 13 ppm of narasin resulted in a reduced Ac:Pr ratio and rumen ammonia when compared to animals supplemented with 20 ppm.

## INTRODUCTION

The use of ionophores, such as monensin and narasin, in animal nutrition has been under public scrutiny and its utilization for growth promotion has been banned in E.U. since 2006 (Clark et al., 2012). The main factor leading to these actions are the concerns related to antimicrobial resistance and the subsequent transfer of the resistant genes from animals to humans (Fajt, 2007). Nonetheless, the Veterinary Feed Directive (VFD) launched by the United States in January 2017 still guarantees the utilization of ionophores for growth promotion and therapeutic action, when approved on the label.

Guan et al. (2006) reported that supplementation with monensin to cattle consuming low- or high-concentrate diets improved feed efficiency (FE), but the reduction in enteric methane (CH_4_) production and protozoal inhibition lasted for a short period of time. Therefore, it was suggested that the inhibitory effect of monensin on ruminal methanogenesis is not persistent due to an adaptation of rumen microbiome to the ionophore (Johnson et al., 1997). Conversely, Odongo et al. (2007) reported that 6 mo supplementation with monensin to dairy cattle consistently decreased enteric CH_4_ production. Daily (200 mg) monensin supplementation for 10 wk reduced CH_4_, acetate: propionate ratio, and increased propionate in beef steers, indicating the efficacy of monensin in modulating the rumen microbiome profile for an extended period of time (Bell et al., 2017a). To the best of our knowledge, no data evaluated the effects of long-term supplementation with narasin on rumen fermentation characteristics of beef cattle. Based on this rationale, we hypothesized that long-term supplementation with narasin to *Bos indicus* steers would lead to transitional and persistent changes on rumen fermentation characteristics. Hence, our objective was to evaluate the effects of long-term supplementation of narasin on rumen fermentation characteristics of *B. indicus* steers receiving a forage-based diet.

## MATERIALS AND METHODS

This study was conducted at the University of São Paulo, Piracicaba campus (USP/ESALQ; Piracicaba, SP, Brazil; 22°43′31″S, 47°38′51″W, and 524 m elevation) from December 2016 to May 2017. All animals used in the present study were cared for in accordance with acceptable practices and experimental protocols reviewed and approved by the ESALQ/USP Institutional Animal Care and Use Committee (IACUC # 2093090119).

### Animals, Housing, and Diets

On day 0 of the study, 30 rumen-cannulated Nellore steers [initial body weight (BW) 281 ± 21 kg] were assigned to individual pens (concrete-surface; 2 × 2 m) in a randomized complete block design according to their initial shrunk BW. Within blocks (*n* = 10), animals were randomly assigned to one of the three treatments: 1) forage-based diet without narasin (CONT; *n* = 10), 2) CONT diet plus 13 ppm of narasin (Zimprova; Elanco Animal Health, São Paulo, SP, Brazil; 13NAR; *n* = 10), and 3) CONT diet plus 20 ppm of narasin (Zimprova; Elanco Animal Health; 20NAR; *n* = 10). The forage offered to the animals throughout the experimental period was Tifton-85 haylage (*Cynodon dactylon* spp.), whereas the vehicle for narasin supplementation was a 50:50 mixture of soybean hull:corn (SBH:C; 25 g of each ingredient, as-fed basis). Additionally, animals (*n* = 10) from the CONT group also received the SBH:C mixture, without the inclusion of narasin. The supplement (SBH:C ± narasin) was offered on a daily basis prior to hay feeding so that the small amount of supplement would not be mixed with hay and compromise the immediate intake of the mixture. The nutritional profile of the forage used in the present experiment is described in Table 1.

**Table 1.** The nutritional profile of the Tifton-85 (*Cynodon dactylon* spp.) haylage used in the present study^1^

		% DM					
Day of the study	DM (%)	CP	NDF	HC^2^	ADF	Ash	EE
0	36.1	12.4	71.0	38.0	33.0	5.9	2.1
28	45.1	8.7	71.2	34.6	36.6	7.2	1.7
56	51.7	6.7	72.5	38.4	34.1	6.0	1.6
84	40.7	6.9	60.5	29.8	30.7	6.8	1.7
112	33.7	9.1	68.2	32.0	36.2	8.1	1.6
140	47.0	7.6	64.4	33.5	30.9	5.9	1.9

DM = dry matter, CP = crude protein, NDF = neutral detergent fiber, HC = hemicellulose, ADF = acid detergent fiber, and EE = ether extract.

^1^Forage was offered in amounts to ensure ad libitum consumption throughout the experimental period (day 0 to 140). Samples were collected every 28 d for nutrient composition determination.

^2^Calculated as: HC = NDF − ADF.

The experimental period lasted 140 d and was divided into five periods of 28 d each. All animals were fed the treatments once daily (0800 h), followed by haylage feeding (0830 h). All animals were allowed ad libitum access to forage, mineral, and freshwater for the entire 140-d period. Narasin was not included in the mineral supplement in order to ensure that the exact amount, based on the individual forage dry matter intake (DMI), would be offered and consumed by the animals. The mineral supplement (Bellmais; Trouw Nutrition; Mirassol, SP, Brazil) used herein contained 178 g Ca, 60 g P, 17 g S, 135 g Na, 5,000 mg Mg, 650 mg Cu, 500 mg Mn, 2,400 mg Zn, 48 mg I, 38 mg Co, 12 mg Se, and 1,000 (max) mg F.

The initial 13 and 20 ppm inclusion of narasin into the 50:50 SBH:C mixture was based on a 5.0 kg forage DMI. Hence, for animals consuming 5.0 kg of forage, the SBH:C mixture would contain 65 and 100 ppm of narasin for 13NAR and 20NAR, respectively. Throughout the experimental period (day 0 to 140), narasin dosage (13 or 20 ppm) offered to the animals was based on the previous day total DMI, by individually weighing the dose to be administered to each animal enrolled into the 13NAR and 20NAR treatments.

### Sampling

At the beginning (day 0) of the experimental period, individual shrunk BW was recorded after 16 h of feed and water withdrawal to determine animal initial BW and to perform the randomization of the animals into blocks and treatments. Throughout the experimental period (day 0 to 140), forage, supplement, and total DMI were recorded daily by collecting and weighing feed refusals (forage only), whereas mineral salt intake was recorded on a weekly basis. Samples of the offered and nonconsumed forage were collected daily from each pen and dried for 48 h at 50 ± 5 °C in forced-air ovens for dry matter (DM) calculation, whereas forage samples were analyzed every 28 d for determination of the nutritional profile (Table 1).

Blood samples were collected via jugular venipuncture into commercial blood collection tubes (Vacutainer, 10 mL; Becton Dickinson, Franklin Lakes, NJ) containing 158 United States. Pharmacopeia units of freeze-dried sodium heparin for plasma collection. All blood samples were placed immediately on ice, subsequently centrifuged (2,500 × *g* for 30 min at 4 °C) for plasma harvest, and stored at –80 °C on the same day of collection. Blood samples were collected on day 0 (immediately prior to the beginning of the experimental period and first treatment offer), 28, 56, 84, 112, and 140 of the experimental period. Samples obtained from day 28 to 140 were collected approximately at 6 h after the SBH:C mixture feeding for plasma urea and glucose determination. Plasma concentration of urea and glucose were determined according to procedures described by Chaney and Marbach (1962) with the adaptations for an ELISA reader (550 nm absorbance; BIO-RAD; Hercules, CA).

Concurrently with the blood sampling, ruminal fluid samples were collected (approximately 100 mL) by squeezing the ruminal contents into 4 layers of cheesecloth and the ruminal fluid pH was immediately determined (Digimed-M20; Digimed Instrumentação Analítica; São Paulo, SP, Brazil). Approximately 50 mL of the ruminal fluid were collected and stored (−20°C) for subsequent analysis of rumen ammonia and molar proportions of individual volatile fatty acids (VFA; acetate, propionate, butyrate, isobutyrate, valerate, isovalerate), as well as the acetate:propionate (Ac:Pr), acetate+butyrate:propionate (AcBu:Pr) ratios, and total VFA. Frozen ruminal samples were prepared for analysis by thawing, centrifuging (15,000 × *g*) for 10 min at room temperature and analyzed for VFA and rumen ammonia according to procedures described by Ferreira et al. (2016) and Broderick and Kang (1980), respectively.

### Total Tract Apparent Nutrient Digestibility

From day 23 to 27 (period 01), 51 to 55 (period 02), 79 to 83 (period 03), 107 to 111 (period 04), and 135 to 139 (period 05), fecal samples were manually collected for apparent nutrient digestibility analysis. The total fecal material was weighed, sampled (approximately 10% of wet weight), and stored at 18 °C for subsequent laboratory analysis. Frozen samples were thawed and dried in a forced air-oven at 55 °C for 96 h. Forage (offer and orts) and fecal samples were ground into a 1-mm screen using a Willey mill (Marconi Equipamentos Laboratories, Piracicaba, SP, Brazil). Dry matter composition was determined by drying the samples in an oven at 105 °C for 24 h and ash content was determined by burning the samples in a muffle furnace at 550 °C for 4 h (AOAC, 1997). Total nitrogen (N) determination was performed using a Leco FP-528 (Leco Corporation; Saint Joseph, MI), according to the methodology proposed by AOAC (1997), whereas neutral detergent fiber (NDF) content was analyzed according to procedures described by Van Soest et al. (1991) with the addition of thermostable α-amylase and sodium sulfite in an Ankom-200 (Ankom Tech Corp., Fairport, NY). Following NDF determination, acid detergent fiber (ADF) was evaluated according to procedures described by Goering and Van Soest (1970) in an Ankom-200 (Ankom Tech. Corp.). Apparent digestibility was calculated according to the formula: TTAD (%) = ((DMI × NCDM) – (FDM × NCFM) × 100) / (DMI × NCDM), where TTAD = total tract apparent digestibility, DMI = dry matter intake, NCDM = nutrient content of the DMI (%), FDM = fecal dry matter, and NCFM = nutrient content of the fecal DM (%).

### Statistical Analysis

For all the variables analyzed herein, animal was considered the experimental unit and all the data were analyzed using the PROC MIXED procedure of SAS (Version 9.4; SAS Inst. Inc.; Cary, NC) and the Satterthwaite approximation to determine the denominator df for the test of fixed effects. For the analysis of all the variables, the model statement contained the effects of treatment, period or day, block, and the treatment × day or period and treatment × block interactions. Data were analyzed using animal as the random variable, whereas the specified term for the repeated statement was day, the subject was animal(treatment), and the covariance structure was first-order autoregressive, which provided the best fit for these analyses according to the smallest Akaike Information Criterion. With the exception of forage DMI and mineral supplement intake, values obtained on day 0 of the study were used as covariates. Additionally, orthogonal contrasts were used to partition specific treatment effects: 1) Supplementation effect: CONT vs. NAR, and 2) Dose effect: 13NAR vs. 20NAR.

Results are reported as least square means and were separated using the PDIFF structure of SAS (SAS Inst. Inc.), as well as covariately adjusted for values obtained on day 0. For all the data, significance was set at *P* ≤ 0.05 and tendencies were denoted if *P* > 0.05 and *P* ≤ 0.10. Results are reported according to the main effects if no interactions were significant.

## RESULTS

For all the variables analyzed herein, no treatment × block interactions were observed (*P* > 0.17) and, therefore, these data will not be presented throughout the manuscript.

### Intake

No treatment effects were observed for forage (*P* = 0.86), concentrate (*P* = 0.36), or total DMI (*P* = 0.23), indicating that narasin administration did not impair any of these parameters (Table 2). Additionally, no differences were observed (*P* > 0.29) on forage intake (g/kg BW), as well as NDF intake (kg or g/kg BW; Table 2). Similarly, no narasin dose effects were observed for any of the aforementioned parameters (*P* > 0.15; Table 2). Additionally, no treatment effects (*P* = 0.22) were detected for weekly mineral DMI, but 13NAR animals tended to have a greater mineral intake compared to 20NAR cohorts (*P* = 0.08; Table 2).

**Table 2.** Forage, concentrate, and total dry matter intake (DMI), as well as mineral salt intake of *Bos indicus* steers receiving a high-forage (*Cynodon dactylon* spp.) diet and supplemented or not (CONT; *n* = 10) with 13 (13NAR; *n* = 10) or 20 (20NAR; *n* = 10) ppm of narasin (Zimprova; Elanco Animal Health, São Paulo, SP, Brazil) throughout the experimental period^1^

	Treatments				*P-*Value^2^				
Item	CONT	13NAR	20NAR	SEM	T	P	T × P	CONT vs. NAR	13NAR vs. 20NAR
Intake^3^									
Forage, kg	6.31	6.37	6.24	0.156	0.86	<0.0001	0.27	0.96	0.59
Forage g/kg BW	1.94	1.98	1.93	0.015	0.91	<0.0001	0.29	0.96	0.60
NDF, kg	4.29	4.33	4.24	0.026	0.88	<0.0001	0.32	0.93	0.63
NDF, g/kg BW	1.32	1.35	1.31	0.010	0.92	<0.0001	0.34	0.97	0.62
Supplement,^4^ g	58.6	60.0	57.1	1.39	0.36	<0.0001	0.78	0.98	0.15
Total, kg	6.37	6.43	6.30	0.176	0.23	<0.0001	0.27	0.34	0.16
Mineral intake,^5^ g	142.6	156.4	120.6	14.51	0.22	<0.0001	0.56	0.82	0.08

^1^Treatments were offered on a daily basis throughout the experimental period (day 0 to 140). Forage was offered in amounts to ensure ad libitum consumption throughout the experimental period (day 0 to 140).

^2^T = treatment effect; P = period effect; T × P = treatment × period interaction; CONT vs. NAR = unsupplemented vs. narasin-supplemented animals; 13NAR vs. 20NAR = dose effect of 13 vs. 20 ppm of narasin.

^3^Forage and concentrate DMI were measured on a daily basis throughout the experimental period (day 0 to 140).

^4^Supplement = 50:50 mixture of soybean hull and corn, containing (13NAR and 20NAR) or not (CONT) narasin.

^5^Mineral intake was measured on a weekly basis.

### Rumen Fermentation Characteristics

Values obtained on day 0 of the study were significant covariates (*P* ≤ 0.03) for rumen concentrations of acetate, propionate, isobutyrate, butyrate, isovalerate, Ac:Pr, and the AcBu:Pr ratio, but did not differ among treatments (*P* > 0.39; data not shown), demonstrating that animals were under a similar management prior to the beginning of the present study. In general, narasin supplementation increased (*P* ≤ 0.03) propionate and total VFA, while decreasing rumen acetate, butyrate, as well as Ac:Pr, and AcBu:Pr ratios (Table 3). Moreover, supplementation with 13 ppm of narasin increased rumen propionate, and decreased butyrate, Ac:Pr and AcBu:Pr ratios compared with 20NAR cohorts (*P* ≤ 0.01; Table 3).

**Table 3.** Rumen volatile fatty acids (VFA) concentrations and pH of *Bos indicus* steers receiving a high-forage (*Cynodon dactylon* spp.) diet and supplemented or not (CONT; *n* = 10) with 13 (13NAR; *n* = 10) or 20 (20NAR; *n* = 10) ppm of narasin (Zimprova; Elanco Animal Health, São Paulo, SP, Brazil) throughout the experimental period^1^

	Treatments			*P-*Value^2^				
Item	CONT	13NAR	20NAR	SEM	T	D	T × D	CONT vs. NAR	13NAR vs. 20NAR
VFA, mM/L									
Acetate	74.21^b^	72.71^a^	72.83^a^	0.163	< 0.0001	< 0.0001	0.12	< 0.0001	0.60
Propionate	13.83^a^	15.82^c^	15.22^b^	0.131	< 0.0001	< 0.0001	0.24	< 0.0001	< 0.01
Isobutyrate	0.81	0.79	0.83	0.018	0.27	< 0.0001	0.92	0.90	0.11
Butyrate	8.89^b^	8.54^a^	8.86^b^	0.073	< 0.01	< 0.0001	0.19	0.04	< 0.01
Isovalerate	1.20	1.20	1.28	0.039	0.32	< 0.0001	0.70	0.49	0.18
Valerate	0.98	0.95	1.00	0.028	0.46	< 0.0001	0.94	0.75	0.23
Total VFA	90.02^a^	97.62^b^	102.08^b^	2.765	0.03	< 0.0001	0.97	0.01	0.25
Ac:Pr	5.40^c^	4.63^a^	4.81^b^	0.048	< 0.0001	< 0.0001	0.41	< 0.0001	0.01
AcBu:Pr	6.04^c^	5.17^a^	5.39^b^	0.049	< 0.0001	< 0.0001	0.48	< 0.0001	< 0.01
Rumen pH	6.70	6.72	6.85	0.065	0.25	< 0.0001	0.33	0.33	0.17
Ammonia, mg/dL	8.38^b^	5.67^a^	5.44^a^	0.621	< 0.01	< 0.0001	0.28	< 0.01	0.80

^1^Treatments were offered on a daily basis throughout the experimental period (day 0 to 140). Rumen samples were collected on day 0 (prior to first treatment administration), 14, 28, 56, 84, 112, and 140 of the study, approximately at 0600 h after treatment administration. Rumen ammonia and pH were evaluated every 28 d. Letters within the same line denote differences at the *P* ≤ 0.05 level.

^2^T = treatment effect; D = day effect = T × D = treatment × day interaction; CONT vs. NAR = unsupplemented vs. narasin-supplemented animals; 13NAR vs. 20NAR = dose effect of 13 vs. 20 ppm of narasin.

A treatment effect was observed (*P* < 0.01) on rumen ammonia concentrations (Table 3). Values obtained on day 0 were not significant covariates (*P* = 0.17) and did not differ among treatments (*P* = 0.94; 8.6, 8.4, and 7.9 mg/dL for CONT, 13NAR, and 20NAR, respectively; SEM = 1.44). Throughout the experimental period, narasin-supplemented animals had reduced ammonia concentrations when compared with CONT cohorts (*P* < 0.01), whereas no differences were observed between 13NAR and 20NAR (*P* = 0.80; Table 3). Furthermore, no treatment effects were observed on ruminal pH (*P* = 0.28; Table 3).

**Figure 1.**
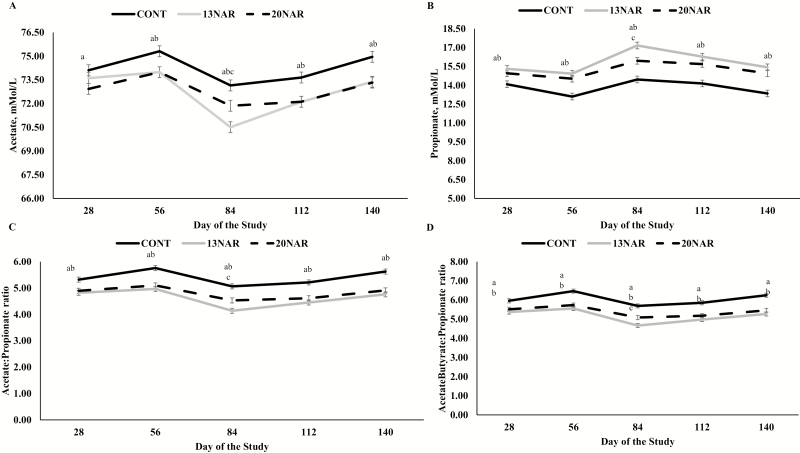
Long-term effects of narasin supplementation on rumen acetate (1-A), propionate (1-B), Acetate:Propionate (Ac:Pr; 1-C), and AcetateButyrate:Propionate (AcBu:Pr; 1-D) ratio of *Bos indicus* steers receiving a high-forage (*Cynodon dactylon* spp.) diet and supplemented or not (**CONT**; *n* = 10) with 13 (**13NAR**; *n* = 10) or 20 (**20NAR**; *n* = 10) ppm of narasin (Zimprova; Elanco Animal Health, São Paulo, SP, Brazil) throughout the experimental period. Treatments were offered on a daily basis throughout the experimental period (day 0 to 140). Rumen samples were collected on day 0 (prior to first treatment administration), 28, 56, 84, 112, and 140 of the study, approximately at 0600 h after treatment administration. Results were covariately adjusted to values obtained on d 0 of the study. For all graphs below, within days, letters indicate the following differences: a = CONT vs. 13NAR (*P* < 0.01); b = CONT vs. 20NAR (*P* ≤ 0.02); c = 13NAR vs. 20NAR (*P* < 0.01).

Throughout the experimental period, narasin-supplemented animals had reduced rumen acetate, Ac:Pr, and AcBu:Pr ratios, as well as greater (*P* ≤ 0.02) rumen propionate concentrations compared with CONT cohorts (Figure 1AD). Although no treatment × day interactions were observed in any of these parameters (*P* > 0.12; Table 3), these effects were reported in figures to support the statement that long-term narasin supplementation did change and maintained these changes on rumen fermentation characteristics of *B. indicus* steers offered a high-forage diet.

In the present study, no treatment effects were observed (*P* > 0.45) on DM, organic matter (OM), crude protein (CP), NDF, ADF, and mineral apparent digestibilities (Table 4). Moreover, no dose effects were observed in any of the digestibility parameters evaluated herein (*P* > 0.23; Table 4).

**Table 4.** Total nutrient apparent digestibility and rumen ammonia concentrations of *Bos indicus* steers receiving a high-forage (*Cynodon dactylon* spp.) diet and supplemented or not (CONT; *n* = 10) with 13 (13NAR; *n* = 10) or 20 (20NAR; *n* = 10) ppm of narasin (Zimprova; Elanco Animal Health, São Paulo, SP, Brazil) throughout the experimental period^1^

	Treatments				*P-*Value^2^				
Item	CONT	13NAR	20NAR	SEM	T	P	T × P	CONT vs. NAR	13NAR vs. 20NAR
Digestibility*,%*									
Dry matter	59.6	59.0	59.6	0.64	0.73	<0.0001	0.67	0.74	0.48
Organic matter	62.6	62.4	62.8	0.51	0.82	< 0.0001	0.57	0.97	0.54
Crude protein	55.0	54.8	55.8	1.07	0.76	<0.0001	0.72	0.81	0.49
Neutral detergent fiber	70.0	69.4	70.0	0.62	0.73	<0.0001	0.88	0.71	0.49
Acid detergent fiber	68.8	70.0	69.1	0.63	0.45	<0.0001	0.79	0.70	0.23
Mineral	24.2	22.7	24.5	2.13	0.83	<0.0001	0.62	0.82	0.56

^1^Treatments were offered on a daily basis throughout the experimental period (day 0 to 140). ^2^Samples for nutrient digestibility were collected from d 23 to 27 (period 01), 51 to 55 (period 02), 78 to 83 (period 03), 107 to 111 (period 04), and 135 to 139 (period 05) of the experimental period. ^3^Letters within the same line denote differences at the *P* ≤ 0.05 level.

^2^T = treatment effect; P = period effect = T × P = treatment × period interaction; CONT vs. NAR = unsupplemented vs. narasin-supplemented animals; 13NAR vs. 20NAR = dose effect of 13 vs. 20 ppm of narasin.

### Blood Parameters

No treatment effects were detected (*P* = 0.73) on mean plasma glucose concentrations (Table 5). Values obtained on day 0 were significant covariates (*P* = 0.04), but did not differ among treatments (*P* = 0.45; 62.9, 60.7, and 62.7 mg/dL for CONT, 13NAR, and 20NAR, respectively; SEM = 1.31). In agreement with rumen ammonia data, a treatment effect tended to be (*P* = 0.08) detected on mean plasma urea concentrations (Table 5). Values obtained on day 0 were not significant covariates (*P* = 0.25) and did not differ among treatments (*P* = 0.54; 21.9, 21.3, and 21.0 mg/dL for CONT, 13NAR, and 20NAR, respectively; SEM = 0.58). Animals fed 13NAR had a reduced mean plasma urea concentration when compared to CONT (*P* = 0.03), whereas no further differences were observed between CONT and 20NAR (*P* = 0.12), as well as 13NAR and 20NAR (*P* = 0.53; Table 5).

**Table 5.** Plasma glucose and urea concentrations of *Bos indicus* steers receiving a high-forage (*Cynodon dactylon* spp.) diet and supplemented or not (CONT; *n* = 10) with 13 (13NAR; *n* = 10) or 20 (20NAR; *n* = 10) ppm of narasin (Zimprova; Elanco Animal Health, São Paulo, SP, Brazil) throughout the experimental period^1^

	Treatments				*P-*Value^2^				
Item	CONT	13NAR	20NAR	SEM	T	D	T × D	CONT vs. NAR	13NAR vs. 20NAR
Glucose, mg/dL	68.6	70.0	68.7	1.37	0.73	< 0.0001	0.73	0.64	0.51
Urea, mg/dL	16.2^b^	13.5^a^	14.2^ab^	0.81	0.08	< 0.0001	0.81	0.03	0.53

^1^Treatments were offered on a daily basis throughout the experimental period (day 0 to 140). Blood samples were collected on day 0 (prior to first treatment administration), 28, 56, 84, 112, and 140 approximately at 0600 h after treatment administration. Letters within the same line denote differences at the *P* ≤ 0.05 level.

^2^T = treatment effect; D = day effect = T × D = treatment × day interaction; CONT vs. NAR = unsupplemented vs. narasin-supplemented animals; 13NAR vs. 20NAR = dose effect of 13 vs. 20 ppm of narasin.

## DISCUSSION

The primary goal of the present study was to evaluate the effects of long-term supplementation (140 d) with narasin on rumen fermentation characteristics of *B. indicus* steers consuming a warm-season, high-forage diet. This hypothesis arose from studies available in the literature reporting a long-term persistent effect (Odongo et al. 2007; Bell et al., 2017a) or a short-term effect (Johnson et al., 1997; Guan et al., 2006) of ionophores on rumen fermentation characteristics of cattle, which in turn, reflects the rumen microbiome of the animals. Moreover, it is important to mention that all of the aforementioned studies evaluated the feeding of monensin as the ionophore; to the best of our knowledge, no other research has studied the effects of long-term supplementation with narasin to beef steers consuming a forage-based diet. In fact, the number of research reports evaluating narasin supplementation to beef cattle is still scarce. Therefore, several discussion points will be focused on monensin and possible similar/different responses upon narasin supplementation.

The lack of effects on forage and mineral DMI observed herein is in agreement with Silva et al. (2015), who reported that inclusion of narasin (13 ppm) into a mineral mixture did not impact mineral and forage DMI. Moreover, Cappellozza et al. (2019) also demonstrated that supplementation with 13 ppm of narasin did not impact the intake of mineral salt and a low-intake, protein-energy supplement (1.7 g/kg BW) in grazing *B. indicus* bulls. Nonetheless, increasing the narasin dose by approximately 50% did not impact concentrate, forage, and total DMI, but tended to decrease mineral supplement intake compared to animals consuming the recommended label dosage (13 ppm). Polizel et al. (2016a) also did not observe differences in forage DMI as a dose of narasin increased from 8 to 32 ppm in wethers fed a high-forage diet. Others have reported effects of ionophore dosage (i.e., monensin) on average daily gain (ADG), FE, and DMI in animals offered a high-concentrate diet (Goodrich et al., 1984; Duffield et al., 2012), and the same was observed in animals offered a high-forage diet for ADG and FE, but not DMI (Bretschneider et al., 2008). In agreement to our results, Ellis et al. (1984) suggested that monensin supplementation often reduces forage DMI in cattle fed high-quality forages [> 65% organic matter digestibility], likely due to the increased metabolic efficiency and ruminal propionate production, whereas in medium- to low-quality forages, gut fill and passage rate might be the limiting factors for additional DMI in a manner that monensin itself does not induce any further negative effect on this parameter. Deswysen et al. (1987) pointed out that the effect of ionophores on DMI varies greatly among animals so that numerous animals would be required to detect significant differences in this scenario. Furthermore, the literature is scarce concerning the effects of DMI and ionophores for cattle offered high-forage warm-season diets, as well as the relationship (if any) between forage quality, forage type (cool- or warm-season), animal DMI, and ionophores (Bretschneider et al., 2008).

The effects of ionophores (monensin and narasin) on nutrient digestibility are variable. In a literature review, Spears (1990) reported that monensin increased OM, DM, and CP digestibility by 2.1, 3.5, and 4.8% compared to a nonsupplemented group, respectively. However, most of the studies evaluated by Spears (1990) offered a high-concentrate diet and/or cool-season forages to the animals and, therefore, these may not reflect the feeding management adopted herein. In agreement with our data, Bell et al. (2017a) reported no differences in DM, OM, and NDF digestibility of beef steers receiving a forage-based diet with or without monensin. Moreover, Polizel et al. (2016b) reported that narasin supplementation to wethers fed a low-quality high-forage diet did not impact DM and OM digestibility, but NDF digestibility increased as the dose of narasin increased (linear effect). The forage used in the study of Polizel et al. (2016b) was lower in quality when compared to the one used herein (6.8 vs. 8.6% CP; 50.4 vs. 70.0% NDF digestibility, respectively) and one might speculate that these differences would likely impact forage intake, rumen retention time, passage rate, and consequently, forage nutrient digestibility.

In the rumen, ionophores modulate this environment by targeting and altering bacterial metabolism of some gram-positive bacteria, such as cellulolytic, proteolytic, and lactate-producing species (Dinius et al., 1976; Richardson et al., 1976; Dennis et al., 1981), as well as protozoa that generate hydrogen ions (Russell, 1987). Several reports in the literature suggest that the effects on methanogenic bacteria following monensin feeding might be indirect, in which hydrogen ions become limited in the rumen and methanogenic bacteria do not have enough substrate for CH_4_ production (Russell, 1987). The combination of these factors will lead to a greater propionate production, as well as a reduced acetate and Ac:Pr ratio, which, in turn, will improve the energetic efficiency of the animals offered ionophores (McGuffey et al., 2001). Nonetheless, DMI and the nutrient composition of experimental diets, monensin dose, and length of monensin treatment period directly impact the rumen fermentation characteristics and/or production parameters (Beauchemin et al., 2008; Duffield et al., 2012; Ellis et al., 2012). Among ionophores evaluated under an in vitro setting, Nagaraja et al. (1987) reported that narasin was more potent than other compounds (monensin, lasalocid, and salinomycin) in manipulating ruminal fermentation characteristics.

To the best of our knowledge, this is the first research study evaluating the effects of increasing doses of narasin on rumen fermentation characteristics of *B. indicus* steers receiving a medium-quality, warm-season forage diet. Considering that ruminal fermentation characteristics often translates what might be observed in terms of performance of the herd, assumptions can be inferred regarding these points. In the present study, narasin supplementation reduced rumen acetate and butyrate and increased rumen propionate and total VFA vs. unsupplemented cohorts. Additionally, dose effects were observed on rumen propionate, Ac:Pr, and AcBu:Pr ratios. In partial agreement to our results, Polizel et al. (2016a) reported a positive linear effect of increasing doses of narasin on total VFA for wethers offered a high-forage diet. In another study, these authors (Polizel et al., 2017) reported no improvements on performance of grazing animals offered a mineral salt containing 13 or 20 ppm of narasin, whereas both resulted in greater performance vs. unsupplemented cohorts. The specific reasons for these results might be related to the curvilinear response often observed when feeding ionophores to grazing beef animals (Bretschneider et al., 2008), in which the optimum narasin dosage could range between 13 and 20 ppm. Nonetheless, no other research study has evaluated the effects of increasing doses of narasin on rumen fermentation characteristics and/or animal growth parameters and additional studies are warranted to evaluate these points.

The lack of effects on rumen pH measurements was expected, given that no significant amounts of supplements and only roughages were offered to the animals, likely maintaining rumen pH at values that would not impair rumen and cellulolytic bacteria function, as well as reducing the daily pH fluctuation. Supporting this statement, Osborne et al. (2004) suggested that in order for monensin to impact rumen pH, lactate should exceed 5 mM, which was unlikely in the present study. It is important to highlight that ruminal pH values observed in the present study were within the range (6.30 to 6.80) to support and maintain adequate fiber digestion of ruminants (Yokoyama and Johnson, 1988). In agreement with our data, Bell et al. (2017a) also did not observe any effect of monensin supplementation on rumen pH of beef steers offered a high-forage diet. Conversely, Bohnert et al. (2016) observed that monensin supplementation to beef steers consuming low-quality temperate forage with supplementation (3 g/kg BW) had lower rumen pH when compared to unsupplemented cohorts.

One of the most pronounced effects of ionophores (i.e., monensin) is the inhibition of ruminal proteolysis and a subsequent reduction in ammonia synthesis (Goodrich et al., 1984; Rogers et al., 1997). Among the bacterial species impacted in vivo and in vitro, *Peptostreptococcus anaerobius*, *Clostridium sticklandii*, and *C. aminophilum* are highlighted (Russell et al., 1988; Chen and Russell, 1989; Krause and Russell, 1996). In ruminants, rumen ammonia levels below 5 mg/dL often limit microbial growth and ruminal fermentation characteristics (Satter and Slyter, 1974; Slyter et al., 1979), whereas our results demonstrate that narasin supplementation reduces rumen ammonia without negatively affecting the aforementioned rumen parameters. In fact, DM and NDF digestibility were not impacted following narasin supplementation, even though rumen VFA profile was permanently altered. Supporting our data, Lana et al. (2000) reported positive effects of monensin in modulating ruminal protein metabolism under greater ruminal pH values. Conversely, Bell et al. (2017a) demonstrated that 10-wk monensin supplementation did not impact rumen ammonia concentrations of beef steers fed a 13.1% CP forage and 0.91 kg/head of a DDGS supplement.

Plasma urea concentrations usually are positively associated with ruminal ammonia concentrations (Broderick and Clayton, 1997). Additionally, animals from all treatment groups had plasma urea concentrations within (CONT) or close to (13NAR and 20NAR) the optimal plasma urea recommendation for growing cattle (15 to 19 mg/dL; Hammond, 1997). One might also speculate that the reduced plasma urea concentrations in the narasin-supplemented groups might be useful to prevent an excessive excretion of protein through the rumen, given that it is unlikely that medium-quality forages would provide enough energy substrates (i.e., starch and/or nonfiber carbohydrates) to optimize the synchrony of energy and protein utilization by rumen microbes (Hammond, 1997; Hall and Huntington, 2008).

It is speculated that since narasin supplementation increases rumen propionate production, an increase in glucose concentration would also be observed through the increase in hepatic gluconeogenic flux (Duffield et al., 2008). In fact, these later authors (Duffield et al., 2008) reported that monensin supplementation increased plasma glucose concentrations in dairy cattle. However, these results have not been consistent and in the present study, narasin supplementation did not affect plasma glucose concentrations of beef steers offered a high-forage diet. In agreement with our results, Bohnert et al. (2016) also reported no effects of monensin supplementation on plasma concentrations of glucose in beef steers and late-gestating cows consuming low-quality cool-season forage. The reason for this observed variability might be related to the fact that the magnitude of the response on plasma glucose is small and might require a large sample size in order to effectively assess it (Duffield et al., 2008), as well as the form of monensin delivery between studies.

As hypothesized herein, our data demonstrate that long-term supplementation with narasin permanently altered rumen fermentation characteristics of beef steers offered a high-forage diet, given the changes in VFA concentrations observed herein (Figures 1A D). Bell et al. (2017a) also reported that 10-wk supplementation with monensin consistently altered VFA proportion of beef animals. Moreover, in a subsequent study, the same authors (Bell et al., 2017b) reported that the effects of monensin on rumen fermentation last up to 7 d after monensin has been withdrawn from the diet. Similarly, Odongo et al. (2007) reported a consistent reduction in methane production by dairy cows receiving monensin during a 6-mo period. Conversely, other authors have suggested that the effects of ionophores on rumen fermentation characteristics are short-term (Guan et al., 2006) and that the rumen microbiome adapts to these molecules and create a self-defense mechanism, which in turn, might cause a loss of efficacy of the ionophores on rumen and performance parameters. Additionally, other studies are warranted to understand the effects of narasin supplementation on rumen microbiome profile of beef animals.

In summary, narasin supplementation to beef steers offered a high-forage diet did not impact forage, mineral, and total DMI, as well as nutrient digestibility, whereas rumen fermentation characteristics, rumen ammonia, and plasma urea concentrations were positively impacted and lasted throughout the experimental 140-d period. Additionally, 13 ppm of narasin resulted in a reduced acetate:propionate ratio and rumen ammonia when compared to animals supplemented with 20 ppm.

*Conflict of interest statement*: The authors confirm that there is no conflict of interest in the present article.
